# Knowledge, Attitudes, and Practices about Trachoma in Rural Communities of Tigray Region, Northern Ethiopia: Implications for Prevention and Control

**DOI:** 10.1155/2020/3270530

**Published:** 2020-07-25

**Authors:** Hailay Gebretnsae, Nega Mamo, Tesfay Teklemariam, Kiros Fenta, Tesfay Gebrehiwet, Abera Berhe, Fana Gebreselasie, Kiros Demoz

**Affiliations:** Tigray Health Research Institute, Mekelle, Tigray, Ethiopia

## Abstract

**Background:**

Trachoma is a neglected tropical disease which is the leading infectious cause of blindness in the world. Trachoma is one of the major health problems in Tigray Region, Northern Ethiopia. However, knowledge, attitudes, and practices about trachoma are not yet studied in depth. The objective of the study was to assess knowledge, attitudes, and practices on trachoma and its associated factors among rural communities in two districts of Tigay Region, Northern Ethiopia.

**Methods:**

A cross-sectional study was conducted in two districts of Tigray Region, Northern Ethiopia, from May 7–24, 2017. Data were collected on paper based, were entered into Epi Info version 3.5.1, and then exported to SPSS version 21 for analysis. Logistic regression analysis was done to identify factors associated with knowledge, attitudes, and practices.

**Results:**

In this study, a total of 194 respondents were included. The overall level of good knowledge, attitudes, and practices on trachoma was 51%, 49.5%, and 35.6%, respectively. Having ever received health education was significantly associated with good knowledge (adjusted odds ratio (AOR) = 4.10; 95% confidence interval (CI): 1.91–8.79) and attitudes (AOR = 2.10; 95% CI: 1.02–4.25). Moreover, good knowledge was associated with good practices on trachoma prevention and control (AOR = 2.86; 95% CI: 1.46–5.62).

**Conclusion:**

Our study implies that areas with high burden of trachoma need to improve communities' knowledge, attitudes, and practices towards trachoma prevention and control in order to eliminate trachoma as a public health problem. Therefore, health education focused on SAFE strategy should be provided to increase knowledge and changing attitudes that contribute for good practices towards trachoma prevention and control among communities.

## 1. Introduction

Trachoma is a neglected tropical disease which is the leading infectious cause of blindness in the world [1–3]. Trachoma largely affects people in low socioeconomic conditions in the rural areas and usually communities with poor hygiene and environmental sanitation [2]. In 2019, globally, the number of people at risk of trachoma was 142.2 million and it is responsible for the visual impairment of about 1.9 million people. Ethiopia is one of the most trachoma-affected countries which shares about 50.6% of the burden in the world [4, 5].

In 1998, the World Health Organization (WHO) formally adopted the SAFE strategy (surgery, antibiotics, facial cleanliness, and environmental improvements) for the effective prevention and control of trachoma [3]. The target for eliminating trachoma as a public health problem can be achieved if the SAFE strategy is properly applied. However, trachoma still remains one of the major health problems in the world [4].

Several factors are associated with increased risk of trachoma. These include lack of water, poor personal hygiene, and environmental sanitation [6–8]. Furthermore, poor knowledge [9, 10], and unfavorable sociocultural perceptions and poor practices about prevention of trachoma are the main factors in the transmission and sustaining of the infection of trachoma in the communities [11–14].

Trachoma is highly endemic in Tigray Region, Northern Ethiopia, especially in Raya-Alamata and Endamekoni districts (the study area). In 2013, the adjusted prevalence of the clinical sign trachomatous inflammation-follicular (TF) among children aged 1–9 years in the enumeration units encompassing Raya-Alamata and Endamekoni districts was 41.4% and 41.0%, respectively [15]. However, there are no data on knowledge, attitudes, and practices on trachoma prevention and control among rural communities in the two districts so far. Therefore, areas with high burden of trachoma such as Alamata and Endamekoni districts need to assess knowledge, attitudes, and practices and its associated factors of trachoma among rural communities. The findings obtained from the study are of paramount importance for health providers and program managers in planning and implementing to improve prevention and control of trachoma among communities. Moreover, it is useful to policy makers to formulate appropriate interventions for implementation of the SAFE strategy.

## 2. Methods

### 2.1. Study Design and Area

A community based cross-sectional study design was conducted to assess knowledge, attitudes, and practices on trachoma among rural communities in two districts (Raya-Alamata and Endamekoni) of Tigray Region, Northern Ethiopia, from May 7–24, 2017. According to the 2007 census, the total estimated population was 96,132 in Raya-Alamata and 94,635 in Endamekoni districts in 2017. From 2014 to 2018; LIGHT FOR THE WORLD in collaboration with the Tigray Regional Health Bureau (TRHB) had a project to eliminate trachoma as a public health problem in the two districts. The focusing activities of the project were mainly on communities' awareness creation and behavioral change to improve hygiene and environmental sanitation by using different communication channels and health education. The Zithromax MDA campaign was carried out in April 2017 (fourth round) before three-four weeks of the data collection in both districts.

### 2.2. Sample Size Determination

The sample size was calculated using single population proportion formula with the assumption of proportion of had heard about trachoma among communities in rural Amhara Region, Ethiopia, which was 85.6% [16], using 5% margin of error, 95% confidence level, and nonresponse rate of 10%, and the total sample size was 209.

### 2.3. Sampling Procedures

Two districts were purposively included in the study. We randomly selected four kebelles (the smallest administrative unit) by the lottery method from the selected districts (2 kebelles from each district). The list of all Women Development Groups (WDGs) with their respective list of households in each selected kebelle was obtained from the health post WDG registration books, and then six WDGs were selected within each selected kebelle using the lottery method. (The WDG is the smallest community organization unit and consists of groups of 25–30 households residing in a neighborhood). Finally, 209 households in the selected WDGs were enrolled using systematic random sampling technique.

### 2.4. Data Collection Procedures and Data Quality Assurance

A structured questionnaire was adapted from a previous study [16], after reviewed the different related literature. Data were collected by face-to-face interviews and by observations for some practice-related questions through a paper-based questionnaire. We interviewed mothers of households about sociodemographic information, knowledge, attitudes, and practices on trachoma, Zithromax uptake, water, sanitation, and hygiene (WASH), health education, and promotion. Their responses on some practice-related questions were verified by observational technique (availability of latrine and utilization, house compound cleanliness, having separated human and animal dwellings, and solid and liquid waste disposal methods). Six diploma nurses to collect data and two bachelor-degree health officers to supervise the data collection process were recruited. Data collectors and supervisors were trained for two days on the questionnaire, data collection techniques, and the purpose of the study. The pretest was conducted in 5% of respondents (10 households) in two kebelles other than the study sites. Daily completeness and checking for missing data was done by the data collectors and supervisors.

### 2.5. Operational Definition

Knowledge, attitudes, and practices on trachoma were assessed using 12 knowledge-related, 4 attitude-related, and 8 practice-related questions on trachoma. Correct answers were given a score of one, incorrect answers were scored zero, and those who scoring greater than the mean were considered to have good knowledge, attitudes, and practices, respectively.

### 2.6. Ethical Approval

The study protocol was reviewed and approved by the institutional ethical review committee of Tigray Health Research Institutes (reference number: THRI/RM012/2017). The support letter was obtained from the Tigray Regional Health Bureau and the selected district health offices. The respondents were informed about the objective and purpose of the study, and verbal consent was obtained. Confidentiality of the information was assured, and participants were informed that they have the right to withdraw from the interview at any stage.

### 2.7. Data Processing and Analysis

Data were entered into Epi Info version 3.5.1 and then imported to SPSS version 21 for analysis. Data cleaning and editing was carried out before analysis. Knowledge, attitudes, and practices were computed separately, and its total scores were recorded in to dichotomous categorical variables (good vs poor) based on their mean score stated in operational definition. Frequency distribution and percentages were performed using frequency tables. Bivariate logistic regression analysis was done for each outcome variables (knowledge, attitudes, and practices). All variables with a *p* value < 0.25 in the bivariate analysis were included in the multivariable logistic regression model. Finally, variables which had a *p* value with less than 0.05 were considered statistically significant.

## 3. Results

### 3.1. Sociodemographic Characteristics of Study Participants

A total of 194 participants were included in this study, and this made a response rate of 92.8%. The remaining (7.2%) participants were not included due to closed households during the data collection period. From a total respondents, 87 (44.8%) were in the age range of 30–49 years and 112 (57.7%) were currently in the union of marital status. Majority of the respondents, 180 (92.8%), were house wife and 155 (79.9%) were illiterate (Table 1).

### 3.2. Knowledge on Trachoma

Most (89.2%) of respondents had ever heard about trachoma. Above half (54.6%) of respondents knew that trachoma can be transmitted from person to person and answered correctly that trachoma can be transmitted by dirty fingers (53.1%), flies (35.6%), and by using contaminated towel (24.7%). The majority, 164 (84.5%), of respondents knew trachoma as a preventable disease, and 161 (83%) of respondents knew that trachoma can lead to blindness. The mean score of respondents on knowledge was 6.79 ± 3.29 standard deviation (SD). Ninety-nine (51%) of respondents were scored above the mean score and classified as having good knowledge on trachoma (Table 2).

### 3.3. Attitudes on Trachoma Prevention and Control

Most; 185 (95.4%), of participants agreed availability of adequate water is important for trachoma prevention, and 171 (86.1%) of respondents believed taking mass drug administration is important to prevent and control trachoma. Overall, 96 (49.5%) of respondents were classified as having good attitudes on trachoma prevention and control with a mean score of 3.39 ± 0.69 SD (Table 3).

### 3.4. Practices on Trachoma Prevention and Control

The majority, 171 (88.1%), of respondents took mass drug administration, and 149 (76.8%) of households had separated human and animal dwellings. However, of the total 194 households, only 72 (37.1%) were utilizing a latrine and 59 (30.4%) had clean house compounds. Out of the total, 69 (35.6%) of respondents were classified as having good practices towards trachoma prevention and control with a mean score of 4.10 ± 1.40 SD (Table 4).

### 3.5. Factors Associated with Good Knowledge, Attitudes, and Practices towards Trachoma

Factors associated with good knowledge, attitudes, and practices towards trachoma prevention and control were observed. Having ever received health education about trachoma was significantly associated with good knowledge (AOR = 4.10; 95% CI: 1.91–8.79) and good attitudes (AOR = 2.10; 95% CI: 1.02–4.25) (Tables 5 and 6). Moreover, good knowledge on trachoma was significantly associated with good practices towards trachoma prevention and control (AOR = 2.86; 95% CI: 1.46–5.62) after adjusting the other variables (Table 7).

## 4. Discussion

This study assessed knowledge, attitudes, and practices on trachoma in two districts of Tigray Region, Northern Ethiopia. Having ever received health education about trachoma was significantly associated with good knowledge and attitudes about trachoma prevention and control. Furthermore, good knowledge was significantly associated with good practices towards trachoma prevention and control.

In this study, 89.2% of respondents had ever heard about trachoma. This result was almost the same with the reports from Ethiopia (92.6%) and Bangladesh (86%), [17, 18]. However, it is higher than the report in Kenya (65.7%) of participants had heard about trachoma [10]. The discrepancy could be due to the differences in social mobilization activities in different sites and studies methods.

The current study shows that 54.6% of respondents knew trachoma can be transmitted from person to person. The respondents mentioned that trachoma can be transmitted by contaminated fingers, flies, and contaminated towels. Similar studies from Kenya and Ethiopia reported that the most reported mode of trachoma transmission was contact with flies and dirty [10, 19]. The current study results also indicate that the majority (84.5%) of respondents knew trachoma as preventable disease. The participants responded that trachoma can be prevented by using latrine, improve environmental sanitation, not using common towel, and washing hand and face with soap. Having knowledge on washing face, not sharing towels, and environmental sanitation was important on prevention of trachoma infection [11].

Regarding attitudes and practices of respondents in the current study, the majority, 86.1%, of respondents agreed that taking mass drug administration is important to prevent and control trachoma and 88.1% of the respondents took mass drug administration. This result is almost comparable with the other reports from Amhara and Tigray regions, and the coverage of mass drug administration ranged from 76.8% to 93.3% [20–22]. On the other hand, 88.1% of respondents agreed that trachoma can be prevented by utilizing latrine. However, only 37.1% of households were utilizing a latrine. Our finding on latrine utilization was lower than from previous studies in Ethiopia [9, 17]. This difference could be due to differences in the study areas because our study was not included urban communities. Latrine utilization has been considered an important intervention for trachoma prevention and control [23]. It is important to reduce burden of flies and improve environmental sanitation through proper disposal of human excreta [24].

In the current study, health education about trachoma was significantly associated with knowledge on trachoma. Previous interventional studies from Ethiopia and Egypt reported that mothers' knowledge was significantly improved after the implementation of the program based health education [16, 25]. Providing health education about trachoma can increase knowledge on trachoma dramatically [26]. With adequate and appropriate health education, communities may increase knowledge on the cause of trachoma, how it is transmitted, and how the disease is prevented [2].

Health education on trachoma was also a predictor of attitudes on trachoma prevention and control. The odds of good attitudes on trachoma prevention and control among participants who had ever received health education were 2.1 times higher than participants who had not ever received health education about trachoma. Health education can help reducing misconceptions and changing cultural perceptions of communities on trachoma prevention and control [11, 27]. Effective health education is the key to building the favorable attitudes on trachoma prevention and control among communities to achieve the goal of eliminating trachoma as a public health problem [28, 29]. In Ethiopia, health extension workers and WDG leaders are giving health education to communities on personal hygiene, environmental sanitation, and mass drug administration for prevention and control of trachoma [22]. This could be the possible explanation that if mothers have received health education on trachoma, they may feel responsibility and accountability to prevent their children and families from trachoma infection. This could be resulted to have good attitudes on prevention and control of trachoma.

The current study revealed that mothers who have good knowledge on trachoma was significantly associated with good practices on trachoma prevention and control. This is supported by a study from Egypt, reported that knowledge was significantly associated with trachoma prevention and control practices [25]. Another study from Ethiopia also revealed that good knowledge was a predictor of good practices towards childhood blindness prevention [19]. Increasing knowledge of trachoma transmission and prevention (e.g., *F*&*E*-related preventive behaviors) is important to improve practices of trachoma prevention and control [30]. This might be due to the reason that if someone knew benefit of personal hygiene and environmental sanitation, she/he could practice to prevent and control trachoma [1].

### 4.1. Limitation of the Study

The sample size of this study was relatively small and did not consider the design effect to account for the multilevel nature of the sampling design. This may have affected both the precision of the prevalence estimates as well as the statistical inferences of the modeling. Responses on knowledge-, attitude-, and practice-related questions might be influenced by social desirability biases. Correct responses for those are socially acceptable opinions and practices, and incorrect responses for those are socially undesirable opinions and practices. There might be missed some factors that could influence knowledge, attitudes, and practices that were not considered in the model.

## 5. Conclusion and Recommendation

Our study implies that areas with high burden of trachoma need to improve communities' knowledge, attitudes, and practices on trachoma prevention and control in order to eliminate trachoma as a public health problem. Therefore, health education focused on the SAFE strategy should be provided at community level, during mass drug administration, and at health facilities while people are waiting to receive treatment to increase good knowledge and changing cultural perceptions that contribute to behavioral changing and increase good practices towards trachoma prevention and control among communities.

## Figures and Tables

**Table 1:** Sociodemographic characteristics of study participants on knowledge, attitudes, and practices on trachoma in two rural districts of Tigray Region, Northern Ethiopia, 2017 (*N* = 194).

Variables	Numbers	Percent
Age		
18–29 Years	69	35.6
30–49 Years	87	44.8
≥50 Years	38	19.6
Marital status		
Currently in union	112	57.7
Currently not in union^*∗∗*^	82	42.3
Occupation		
House wife	180	92.8
Others^*∗*^	14	7.2
Educational status		
Illiterate	155	79.9
Literate	39	20.1
Family size		
≤5	132	68
>5	62	32
Monthly family income in US dollar		
<22 USD	84	43.3
22–44 USD	87	44.8
>44 USD	23	11.9

^*∗*^Others (government employed, merchant, and daily laborer). ^*∗∗*^Currently not in union (single, widowed, divorce, and separated).

**Table 2:** Knowledge of study participants on trachoma in two rural districts of Tigray Region, Northern Ethiopia, 2017 (*N* = 194).

Variables	Numbers	Percent
Heard about trachoma disease		
Yes	173	89.2
No	21	10.8
Knew trachoma can be transmitted from person to person		
Yes	106	54.6
No	88	45.4
Knew trachoma can be transmitted by contaminated fingers		
Yes	103	53.1
No	91	46.9
Knew trachoma can be transmitted by flies		
Yes	69	35.6
No	125	64.4
Knew trachoma can be transmitted by contaminated towels		
Yes	48	24.7
No	146	75.3
Knew trachoma as preventable disease		
Yes	164	84.5
No	35	18
Knew trachoma can lead to blindness		
Yes	161	83
No	33	17
Knew trachoma can be prevented by washing hands with soap		
Yes	118	60.8
No	76	39.2
Knew trachoma can be prevented by washing face		
Yes	131	67.5
No	63	32.5
Knew trachoma can be prevented by using latrine		
Yes	37	19.1
No	157	80.9
Knew trachoma can be prevented by improving environmental sanitation		
Yes	159	82
No	30	15.5
Knew trachoma can be prevented by not using common towel		
Yes	48	24.7
No	146	75.3
Mean +SD	6.79 ± 3.29	
Median + IQR (interquartile range)	7 ± 5.25	
Score of knowledge on trachoma		
Good	99	51
Poor	95	49

**Table 3:** Attitudes of study participants on trachoma in two rural districts of Tigray Region, Northern Ethiopia, 2017 (*N* = 194).

Variables	Numbers	Percent
Availability of adequate water is important for trachoma prevention and control		
Agree	185	95.4
Disagree	9	4.6
Personal hygiene is important for trachoma prevention		
Agree	134	69.1
Disagree	60	30.9
Latrine utilization is important for trachoma prevention		
Agree	171	88.1
Disagree	23	11.9
Taking mass drug administration is important to prevent and control trachoma		
Agree	167	86.1
Disagree	27	13.9
Mean +SD	3.39 ± 0.69	
Median + IQR	3 ± 1	
Score of attitudes on trachoma prevention and control		
Good	96	49.5
Poor	98	50.5

**Table 4:** Practice of community towards trachoma prevention and control in two rural districts of Tigray Region, Northern Ethiopia, 2017 (*N* = 194).

Variables	Numbers	Percent
Took mass drug administration for prevention of trachoma		
Yes	171	88.1
No	23	11.9
Utilization of adequate water for bathing		
Yes	150	77.3
No	44	22.7
Having separated human and animal dwellings		
Yes	149	76.8
No	45	23.2
Latrine utilization		
Yes	72	37.1
No	122	62.9
Handwashing practice after using latrine		
Yes	95	49
No	99	51
Having proper solid waste disposal management		
Yes	83	42.8
No	111	57.2
Having proper liquid waste disposal management		
Yes	15	7.7
No	179	92.3
Having clean house compound		
Yes	59	30.4
No	135	69.6
Mean +SD	4.10 ± 1.40	
Median + IQR	4 ± 2	
Score of practices on trachoma prevention and control		
Good	69	35.6
Poor	125	64.4

**Table 5:** Logistic regression analysis of selected variables with knowledge of study participants on trachoma in two rural districts of Tigray Region, Northern Ethiopia, 2017 (*N* = 194).

Variables	Knowledge		COR (95% CI)	AOR (95% CI)
	Good (%)	Poor (%)		
Marital status				
Currently in union	53 (53.5)	59 (62.1)	1	1
Currently not in union	46 (46.5)	36 (37.9)	1.42 (0.80–2.52)	1.39 (0.77–2.53)
Availability of radio/television in the households				
Yes	19 (19.2)	12 (12.6)	1.64 (0.75–3.60)	1.35 (0.60–3.03)
No	80 (80.8)	83 (87.4)	1	1
Ever received health education about trachoma				
Yes	88 (88.9)	62 (65.3)	**4.26 (2.00–9.07)** ^*∗∗∗*^	**4.10 (1.91–8.79)** ^*∗∗∗*^
No	11 (11.1)	33 (34.7)	1	1

^*∗*^Statistically significant at 0.05<*p* < 0.01. ^*∗∗*^Statistically significant at 0.01<*p* < 0.001. ^*∗∗∗*^Statistically significant at *p* < 0.001.

**Table 6:** Logistic regression analysis of selected variables with attitude of study participants on trachoma in two rural districts of Tigray Region, Northern Ethiopia, 2017 (*N* = 194).

Variables	Attitude		COR (95% CI)	AOR (95% CI)
	Good (%)	Poor (%)		
Age				
18–29 years	30 (31.2)	39 (39.8)	1	1
30–49 years	49 (51)	38 (38.8)	1.68 (0.89–3.17)	1.46 (0.75–2.84)
≥50 years	17 (17.3)	21 (21.4)	1.10 (0.47–2.34)	1.20 (0.53–2.72)
Family size				
≤5	59 (61.5)	73 (74.5)	1	1
>5	37 (38.5)	25 (25.5)	1.83 (0.99–3.38)	1.52 (0.78–2.93)
Ever received health education about trachoma				
Yes	81 (84.4)	69 (70.4)	**2.27 (1.13–4.58)** ^*∗*^	**2.10 (1.02–4.25)** ^*∗*^
No	15 (15.6)	29 (29.6)	1	1

^*∗*^Statistically significant at 0.05<*p* < 0.01. ^*∗∗*^Statistically significant at 0.01<*p* < 0.001. ^*∗∗∗*^Statistically significant at *p* < 0.001.

**Table 7:** Logistic regression analysis of selected variables with practice of communities on trachoma in two rural districts of Tigray Region, Northern Ethiopia, 2017 (*N* = 194).

Variables	Practice		COR (95% CI)	AOR (95% CI)
	Good (%)	Poor (%)		
Marital status				
Currently in union	33 (47.8)	79 (63.2)	1	1
Currently not in union	36 (52.2)	46 (36.8)	**1.87 (1.03–3.40)** ^*∗*^	1.71 (0.88–3.23)
Educational status				
Illiterate	52 (75.4)	103 (82.4)	1	1
Literate	17 (24.6)	22 (17.6)	1.53 (0.75–3.13)	1.01 (0.43–2.40)
Occupation				
House wife	61 (88.4)	119 (95.2)	1	1
Others	8 (11.6)	6 (4.8)	2.60 (0.86–7.83)	2.10 (0.57–7.64)
Availability of radio/television in the household				
Yes	18 (26.1)	13 (10.4)	**3.04 (1.39–6.68)** ^*∗∗*^	2.20 (0.93–5.20)
No	51 (73.9)	112 (89.6)	1	1
Time taken to fetch water from its source				
≤30	57 (82.6)	94 (75.2)	1.57 (0.75–3.29)	0.97 (0.40–2.40)
>30 minutes	12 (17.4)	31 (24.8)	1	1
Access to sustainable sufficient water supply				
Yes	63 (91.3)	104 (83.2)	2.12 (0.81–5.54)	2.02 (0.66–6.18)
No	6 (8.7)	21 (16.8)	1	1
Ever received health education about trachoma				
Yes	60 (87)		**2.59 (1.16–5.78)** ^*∗*^	1.55 (0.63–3.83)
No	9 (13)		1	1
Total score of knowledge on trachoma				
Good	22 (31.9)	73 (58.4)	**3.0 (1.62–5.57)** ^*∗∗*^	**2.86 (1.46–5.62)** ^*∗∗*^
Poor	47 (68.1)	52 (41.6)	1	1
Total score of attitudes on trachoma				
Good	30 (43.5)	68 (54.4)	1.55 (0.86–2.80)	1.51 (0.79–2.87)
Poor	39 (56.5)	57 (45.6)	1	1

^*∗*^Statistically significant at 0.05<*p* < 0.01. ^*∗∗*^Statistically significant at 0.01<*p* < 0.001. ^*∗∗∗*^Statistically significant at *p* < 0.001.

## Data Availability

The datasets used and/or analyzed during the current study are available from the corresponding author on reasonable request.

## Abbreviations

AOR: Adjusted odds ratio
CI: Confidence interval
COR: Crude odds ratio
SD: Standard deviation
WDG: Women developmental group.
